# Development of a New Extraction Method for Pomegranate and Metabolite Profiling by a LC-MS and ^1^H NMR Combined Approach

**DOI:** 10.3390/foods13101429

**Published:** 2024-05-07

**Authors:** Luciana Maria Polcaro, Marzieh Rahmani Samani, Sonia Piacente, Milena Masullo

**Affiliations:** 1 Dipartimento di Farmacia, Università degli Studi di Salerno, Via Giovanni Paolo II, 84084 Salerno, Italy; lpolcaro@unisa.it (L.M.P.); mrahmanisamani@unisa.it (M.R.S.); mmasullo@unisa.it (M.M.); 2 PhD Program in Drug Discovery and Development, Università degli Studi di Salerno, Via Giovanni Paolo II n. 132, 84084 Fisciano, Italy; 3 National Biodiversity Future Center (NBFC), 90133 Palermo, Italy

**Keywords:** green extraction, SLDE-Naviglio, chemical profiles, *Punica granatum*, pomegranate food supplement, LC-MS analysis, NMR analysis

## Abstract

The pomegranate (*Punica granatum* L.) market has steadily grown due to the increasing demand of health-conscious consumers of products with health-promoting effects. Recently, a growing interest in developing ecological and environmentally friendly extraction methods has led to investigating extraction procedures to obtain a higher extraction yield using a lower amount of solvents and energy. Herein, a new extraction procedure was developed to obtain an enriched pomegranate food supplement by using the peels of pomegranate, cultivar “Dente di Cavallo” and its juice. The extraction was performed through a non-conventional extraction technique like SLDE-Naviglio using ethanol and pomegranate juice as a solvent, and peels as a matrix. The extract was analysed by a combined approach based on LCESI/QExactive/MS/MS and NMR analysis, and its chemical profile was compared with those of pomegranate juice and of the extract obtained from peels by SLDE-Naviglio by using ethanol:H_2_O. The LC-MS analysis highlighted the presence of hydrolysable tannins, flavonoids, ellagic acid and phenol glucoside derivatives, while ^1^H NMR analysis completed the profile by detecting the primary metabolites. The LC-MS and ^1^H NMR analysis indicated that the extract obtained by SLDE-Naviglio using ethanol and pomegranate juice was enriched in the bioactives as confirmed by the highest phenolic, tannin and flavonoid content.

## 1. Introduction

Pomegranate (*Punica granatum* L.) is a fascinating fruit with a rich history of traditional uses and a wide range of bioactive compounds that contribute to its health benefits [1]. Pomegranates are rich in various bioactive constituents, and more than 500 compounds have been identified from different parts of the pomegranate tree; some of the major compounds include ellagitannins, gallotannins, anthocyanins, flavanol–anthocyanin adducts, flavonoids, phenolic acids, and other phenols [1,2]. The consumption of pomegranate is associated with several health benefits, and the pomegranate juice is widely consumed not only for its pleasant taste but also for its potential health-promoting properties, including efficacy against infectious diseases, atherosclerosis, coronary heart disease and cancer [3]. Also, the pomegranate fruit peel is considered a traditional medicinal herb with applications in treating palpitations, ischemia and bronchitis [1]. Various parts of the pomegranate tree are utilized for their bioactive compounds. These parts include the juice, seeds, peel, flowers, leaves and bark [4,5,6].

Increasing evidence has revealed that pomegranate possesses several pharmacological properties, including its efficacy against infectious diseases, atherosclerosis, coronary heart disease and cancer. Pomegranate extracts blocked nuclear factor kappa B (NF-kB) activity in a prostate cancer model [7] and renal cell carcinoma in vitro [8]. Several studies have proposed the use of pomegranate, and especially its derivative punicalagin, as a potential nutritional strategy in slowing the progression of neurodegenerative disorders; punicalagin inhibits lipopolysaccharide-induced memory impairment via anti-inflammatory and anti-amylogenic mechanisms [9].

Consumption of pomegranate is reported to improve gut microbiota and prevent obesity and diabetes [1]. Pomegranate juice and extracts (400, 100, and 25 mg/mL) showed a prebiotic effect when used in in vitro stool cultures, enhancing the growth of *Bifidobacteria* and *Lactobacilli* while simultaneously inhibiting the growth of the *Bacteroides fragilis* group, *Clostridia* and *Enterobacteriaceae* [10]. In addition, pomegranate juice extract and ellagitannins have been shown to inhibit α-glucosidase activity in vitro and to reduce starch digestibility under simulated gastrointestinal conditions, confirming the great potential of pomegranate juice to improve postprandial hyperglycaemia, which is linked to type II diabetes [11]. 

In vivo and ex vivo studies clearly demonstrated that pomegranate could represent a natural choice for the treatment of different diseases [1]. Several mouse intervention studies have shown that pomegranate extracts decrease inflammation and LDL (low-density lipoprotein) cholesterol in high-fat diet-induced obese mice and can reduce hepatic lipid peroxidation and serum glucose levels in healthy rats, in addition to improving glycaemic control and increased relative beta cell numbers in alloxan-induced diabetic rats [12,13]. 

Pomegranate peel extracts showed a protective effect on oxidative stress in mice loaded with restraint stress, which may be related to its free radical scavenging activity and lipid peroxidation inhibitory effect. The results of in vivo investigations of Middha et al. revealed that pomegranate extract could increase insulin and decrease blood glucose in normal and diabetic rats. They also showed that extract could increase the number of pancreatic β-cells in normal and diabetic rats [14]. In another in vivo study on rats fed with pomegranate peel, it was indicated that pomegranate peel provided protective activity against carbon tetrachloride (CCl4) toxicity, highlighting interesting antioxidant activity [15]. Moreover, pomegranate may be suggested as a potential anti-inflammatory fruit. Houstonab et al., in their ex vivo investigation, indicated that topically applied pomegranate tannins and pomegranate rind extract showed significant anti-inflammatory activity in ex vivo skin on the cyclooxygenase-2 expression [16]. 

Regarding central nervous system pathologies, pomegranate juice reduces amyloid load and improves action in Alzheimer’s disease in the mice model. In one study, Hartman et al. found that there was 50% less amyloid deposition and soluble β-amyloid accumulation in the mice hippocampus treated with pomegranate juice as compared to the control group, which would be considered to improve Alzheimer’s disease [17]. Some components of the pomegranate extract, such as punicalagin and ellagic acid, have the potential to be absorbed and to enter the brain to inhibit microglial action via debilitation of the nuclear factor of activated T-cell activity [18]. 

Based on these considerations, food industries have shown a growing interest in dietary supplements and nutraceuticals based on pomegranate. This is evident from the number of products available on the market, such as fresh juice, jelly, jams, candies, peel powder, face creams, and various other products formulated with pomegranate [19].

Pomegranate juice is obtained from the fruits, after peeling, with natural pressure and without chemicals. In this way, the bioactive compounds are transferred to the juice, which in turn retains the organoleptic properties of the fruits. Considering the occurrence of many bioactive compounds in the peels [20], they can be exploited to enrich the juice further, providing added value to the pomegranate industry [4,21]. 

In the last few years in Italy, especially in the southern regions, there has been a rapid expansion of the areas cultivated with pomegranate [22]. A vast range of pomegranate varieties can be found in Italy. The most widespread commercial cultivars currently grown in Italy are “Wonderful” (originating from Florida), “Acco” (from Israel) and “Dente di Cavallo” (from Italy) [23]. This latter represents one of the most important Italian cultivars because it appears as a versatile and resilient variety, valued for its adaptability to challenging environmental conditions and for its high-quality and flavourful fruit [24]. 

To find a helpful tool for the pomegranate juice industry, to obtain an enriched food supplement and at the same time to apply an effective extraction process, combining a byproduct as the peels with the juice, a new strategy of extraction was developed for *Punica granatum*, cultivar “Dente di Cavallo”. In this way, the pomegranate juice was used as an extraction solvent for the peels, developing a new food supplement and improving its value as a functional product.

In detail, the peels were submitted to a non-conventional extraction technique like solid–liquid dynamic extraction (SLDE-Naviglio) using EtOH and pomegranate juice (70:30). The chemical profile of this extract was analysed by a combined approach based on LCESI/QExactive/MS/MS and NMR analysis and further compared with those of the only pomegranate juice and of the extract obtained from peels by SLDE-Naviglio technique using EtOH:H_2_O (70:30) as solvent. In addition, the total phenolic content, the total flavonoid and the total tannin content of the juice and extracts were evaluated.

## 2. Materials and Methods

### 2.1. Chemicals

The solvent for extractions, LC-MS and NMR analysis, deuterium oxide (D_2_O) plus TSP (4,4-dimethyl-4-silapentane-1-sulfonic acid, sodium dihydrogen phosphate (NaH_2_PO_4_), sodium hydrogen phosphate (Na_2_HPO_4_), sodium azide (NaN_3_), potassium phosphate buffer, potassium persulfate (K_2_S_2_O_8_), Folin–Ciocalteu phenol reagent, polyvinylpolypyrrolidone (PVPP), NaNO_2_, AlCl_3_, NaOH, rutin and gallic acid were purchased from Sigma Aldrich (Milano, Italy).

### 2.2. Sample Preparation and Extraction Procedure

**Figure 1:**
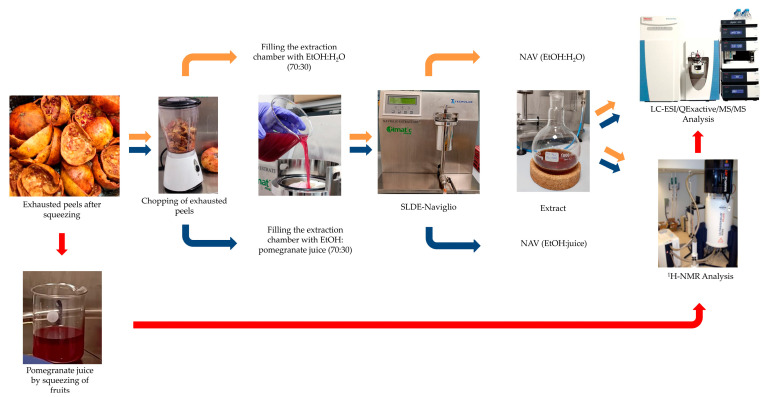
Workflow of the chemical investigation of *P. granatum* cv. “Dente di Cavallo”.

Fresh fruits of *Punica granatum* L., “Dente di Cavallo” cultivar were afforded as a kind gift by Azienda Agricola Giuseppe Rizzo (Paestum, SA, Italy). The enriched extract was obtained from peels, using a mixture of ethanol (70%) and pomegranate juice (30%) as the extraction solvent and SLDE-Naviglio (NAV) as the extractive technique (NAV, EtOH:juice). The fruits were squeezed to obtain the juice; the exhausted peels (Figure 1) were chopped with the shredder and placed in the extraction bag which was then inserted into the extraction chamber (Figure 1). Finally, the mixture of ethanol and pomegranate juice (70:30) was used as an extraction solvent. To compare this innovative extraction strategy using juice instead of water, the same procedure was carried out to extract peels using 70:30 ethanol:H_2_O. SLDE-Naviglio extraction was performed using a Timatic micro series Naviglio extractor, 280.76 g of peels, 800 mL of solvent and an extractive protocol of 20 extractive cycles of 12 min (9 min in the static phase and 3 min in the dynamic phase).

### 2.3. Analysis by LC-ESI/QExactive/MS/MS

Liquid chromatography coupled with electrospray ionization and a high-resolution mass spectrometer (QExactive) was employed for the analysis. A QExactive mass spectrometer, a hybrid Quadrupole-Orbitrap Mass Spectrometer manufactured by Thermo Fischer, was used.

The analysis was conducted in negative ion mode, providing information about negatively charged ions. A Luna C18 column with a particle size of 5 µm and dimensions of 150 × 2.00 mm (Phenomenex) was used. The flow rate during the liquid chromatography separation was 0.2 mL/min. A binary solvent system was employed, consisting of eluent A (water with 0.1% formic acid) and eluent B (acetonitrile with 0.1% formic acid). The elution gradient started at 5% B, increased to 50% in 30 min, reached 95% after 15 min, and then returned to 5% after an additional 5 min. The autosampler injected 5 µL of each extract at a concentration of 1 mg/mL.

ESI source parameters included a capillary voltage of −48 V, tube lens voltage of −176.47 V, ion source temperature of 280 °C, and sheath and auxiliary gas flow rates (N_2_) of 15 and 5, respectively. The full range of *m*/*z* (mass-to-charge ratio) for acquiring MS spectra was set from 150 to 1500. For fragmentation studies, a data-dependent scan was implemented, where precursor ions corresponding to the most intensive peaks were fragmented with a collision energy of 30%. Xcalibur software version 2.2 was utilized for instrument control, data acquisition and data analysis.

### 2.4. ^1^H-NMR Analysis and Data Processing

For ^1^H-NMR analysis, the extracts and juice (each, 6.1 mg) were analysed in triplicate and dissolved in 537 μL of phosphate buffer (1M KH_2_PO_4_, D_2_O, and 2mM NaN_3_ to prevent microbial contamination), and after that, 13 μL of 1mM TSP (trimethylsilyl propanoic acid) was added as internal standard and finally filled into a 5mm NMR tube. All measurements were performed on a Bruker Avance III 600 Ascend NMR spectrometer (Bruker, Germany) operating at 600 MHz. Experiments were run at 300 K in automation mode after loading individual samples on a Bruker Automatic Sample Changer, interfaced with the software IconNMR (Bruker). A zgesgp Bruker standard pulse sequence was applied for each sample to suppress the residual water signal (see Supplementary Materials). The Free Induction Decay (FID) signal data were analysed using a processing module in the Chenomx NMR Suite 10.0 (Chenomx Inc., Edmonton, AB, Canada). The data were automatically zero-filled and underwent Fourier Transformation (FT). The data were then carefully phased, and baseline-distortion correction was carried out. Compounds were identified by matching spectral signals to the Chenomx and Human Metabolome Database (HMDB) 600 MHz libraries. The reference compound TSP was used as an internal standard for the chemical shifts (set to 0 ppm) and as reference signal for the quantification. Data quantification was performed by comparing the integration of a known reference signal (TSP) with the signals derived from a library of compounds containing chemical shifts and peak multiplicities for all of the resonances of the constituents [25]. The results were exported as an Excel file for further analysis. The quantitative results are expressed as means of the standard deviation (SD) of three independent experiments. 

*Punica granatum* extracts and juice were analysed by 2D-NMR experiments too. For the HSQC analysis, a spectral window of 12 ppm and 165 ppm was used for proton and carbon, respectively, with 1 K data points, 64 number scans, increments of 256 t1, and a recycle delay of 2 s. The HMBC was obtained with a spectral window of 12 ppm and 230 ppm for proton and carbon, respectively, with 4 K data points, 128 scans, increments of 256 t1, and a recycle delay of 2 s. 

### 2.5. Determination of Total Phenolic and Total Tannin Content

The total phenolic content of the juice and extracts was determined using the Folin–Ciocalteu assay, as reported by Masullo et al. [26] (see Supplementary Materials). The total tannin content of extracts was determined via polyvinylpolypyrrolidone (PVPP) (see Supplementary Materials). The method involves selectively removing non-tannin phenolics from the extracts using PVPP, followed by determining the total phenolic content in both the original extracts and the supernatant. The total tannin content was then calculated by subtracting the non-tannin phenolics percentage from the total phenolics percentage. This approach helped us to quantify the contribution of tannins to the overall phenolic content in the extracts. In detail, each extract (1 mL) was treated with PVPP (200 mg), mixed with distilled water (2 mL), and vortexed for 10 min. The reaction mass is maintained at 40 °C for 2 h. This indicates that the reaction is temperature-sensitive and requires a specific duration for optimal results. After the reaction, the mixture is subjected to centrifugation, a common technique for separating components based on their density, with denser particles moving to the bottom of the tube. The supernatant, which is the liquid portion remaining after centrifugation, is collected. This portion contains phenolic constituents other than tannins. The collected supernatant is then subjected to the same procedure used for determining the total phenolic content in the extracts.

The total tannin content of the various extracts was calculated as follows:

Tannins (%) = Total Phenolics (%) − Non-Tannin Phenolics (%)

This calculation is based on the premise that the difference between the total phenolics and the phenolics remaining in the supernatant after treatment with PVPP represents the tannin content.

### 2.6. Determination of Total Flavonoid Content

The total flavonoid content was measured using the Allumine Chloride colorimetric assay using rutin as a standard following the procedure previously described [27] (see Supplementary Materials).

## 3. Results and Discussion

### 3.1. Extraction Procedure of Pomegranate

Pomegranate contains several phytochemicals which are qualitatively and quantitatively different in arils and peels. In recent years, there has been a growing interest in developing ecological and environmentally friendly methods for natural product extraction [28,29,30].

For these reasons, selecting an appropriate extraction method and solvent is critical to obtain a “green” extract rich in metabolites. The Solid–Liquid-Dynamic-Extraction (SLDE)-Naviglio, based on a suction effect generated by the compression of the extracting solvent on the solid, was chosen as the extraction technique.

Comparing the new extraction method with other contemporary green extraction technologies, it is possible to observe the several advantages that SLDE-Naviglio offers as solid–liquid extraction. The literature data highlighted how Supercritical Fluid Extraction (SFE) requires a very high pressure, is very expensive and not versatile. Considering Ultrasound-Assisted Extraction (UAE), the problem is related to the poor extract stability and quality because extracted compounds suffer from the direct bombing of cavitation generated by ultrasounds and can undergo transformations resulting in the loss of their beneficial activity [31]. The limits for the Accelerated Solvent Extraction (ASE) are represented by a partial extraction because of the static system and a possible degradation of the active ingredients due to operative conditions (high temperature and pressure). Indeed, SLDE requires a few extraction cycles, about 30 (depending on the matrix), to bring many plant matrices to complete exhaustion [31]. Moreover, SLDE is an inexpensive technique and requires minimal energy expenditure when compared with SFE or ASE, both requiring the use of high temperatures.

In summary, the main advantages of SLDE are as follows: exhaustion in a short time, with solid matrices containing extractable substances at low operating temperatures (environment or sub-environment); reproducibility of the extraction with a guarantee of the production of high-quality extracts, easy use, low-energy consumption, and the speed of the extraction process [31]. Herein, to obtain an enriched food supplement, a new approach of extraction was developed. The peels were extracted by SLDE-Naviglio by using EtOH and pomegranate juice (70:30) as the extraction solvent, to obtain the extract named NAV(EtOH:juice). Moreover, the peels were also extracted by SLDE-Naviglio by using EtOH:H_2_O (70:30) to obtain the extract indicated as NAV (EtOH:H_2_O). To highlight if the new strategy effectively provided a food supplement enriched in bioactive compounds, NAV(EtOH:juice), its chemical profile was compared with those of NAV(EtOH:H_2_O) and pomegranate juice (Figure 1).

### 3.2. LC-ESI/QExactive/MS/MS Analysis

To better visualize the chemical profile of the juice and the extracts, they were subjected to repartition with n-butanol:water (1:1) to remove free sugar. The butanol fractions of the juice and the extracts were analysed by HPLC coupled with electrospray ionization and hybrid Quadrupole-Orbitrap mass spectrometry (LC-ESI/QExactive/MS/MS) in negative ion mode. The accurate masses, characteristic fragmentation patterns, retention times, and the literature data allowed us to putatively identify 17 compounds consisting of ellagitannins, characterised by the presence of a C-C linkage between galloyl units [32], flavonoids, phenol glucoside derivatives, and phenolic acids (Table 1).

According to the typical behaviour of ellagitannins (ETs) [33,34], in most cases, LC-ESI/HRMS spectra of monomeric ETs showed, in addition to negative ions corresponding to the [M−H]^−^, the [M−2H]^2−^ doubly charged molecular ions which corroborated the determination of molecular formula (Table 1). The MS/MS^n^ spectra were highly informative, characterised by the occurrence of product ions diagnostic for ET subclasses. In detail, compound **1** displayed a [M−H]^−^ pseudomolecular ion at *m*/*z* 481.0625, attributed to the HHDP-glucose (hexahydroxyphenoyl-glucose) based on its molecular weight and on the presence in the relative tandem mass spectrum, of an intense product ion at *m*/*z* 300.9991, formed by neutral loss of a glucose unit and corresponding to the ellagic acid anion generated by rearrangement of the HHDP moiety (Table 1) [33,35].

Compound **2** showed a [M-H]^−^ ion at *m*/*z* 781.0542, and in the MS/MS spectrum, product ions at *m*/*z* 600.9899 ascribable to the gallagic acid moiety, at *m*/*z* 575.0115 corresponding to the neutral loss of 25 Da of a carbonyl group, and *m*/*z* 300.9988 corresponding to the ellagic acid moiety.

Both isomers of punicalagin (**5**, **6**) showed in the LC-ESI/HRMS spectra a [M−H]^−^ ions at *m*/*z* 1083.0601 and a doubly charged molecular [M-H]^2−^ ions at *m*/*z* 541.0262. The MS/MS spectra of compounds **5**, **6** were characterised by the presence of product ions at *m*/*z* 600.9899 corresponding to the gallagic acid moiety, *m*/*z* 575.0101 corresponding to the neutral loss of 25 Da of a carbonyl group, *m*/*z* 300.9991 corresponding to the ellagic acid moiety [36,37], and *m*/*z* 275.0199 corresponding to the neutral loss of 25 Da (CO_2_) of carbonyl in the form of carbon dioxide. These characteristic fragmentation patterns, along with their retention time in the LC-ESI/HRMS spectra, allowed us to discriminate the two punicalagin isomers in punicalagin isomer α (**5**) and punicalagin isomer β (**6**) [32].

Compounds **7** displayed a [M-2H]^−^ ion at *m*/*z* 799.0646, and fragment ions at *m*/*z* 300.9991 corresponding to the ellagic acid moiety and at *m*/*z* 273.0043 corresponding to the neutral loss of 27 Da (CO) of a carbonyl group. Based on the above considerations and the literature [38], it was identified as granatin A.

Compound **11** generated [M-H]^−^ ion at *m*/*z* 951.0748, and in MS/MS spectrum, it produced the same fragment ions of granatin A at *m*/*z* 300.9991 and *m*/*z* 273.0043. It was assigned as granatin B [38].

**Figure 2:**
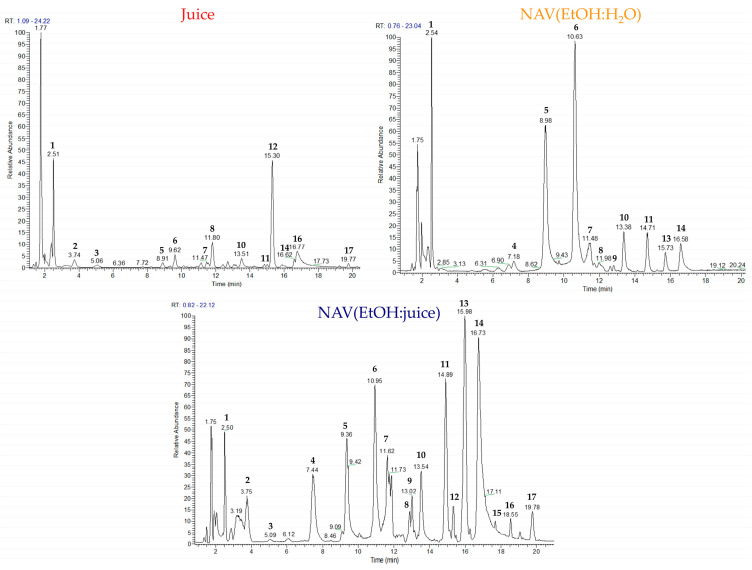
LC-ESI/QExactive/MS Base Peak profiles of *P. granatum* juice, NAV(EtOH:H_2_O) extract and NAV(EtOH:juice) extract.

The LC-MS profiles of butanol fractions of juice and extracts showed some interesting differences (Figure 2). In particular, the butanol fraction of juice was the poorest in metabolites. The butanol fraction of the NAV(EtOH:juice) extract was the richest in metabolites. The most intense peaks observed were ellagic acid (**14**) and its pentose derivative (**13**), followed by granatin B (**11**) and punicalagin α (**5**) and β (**6**). The butanol fraction of the NAV(EtOH:H_2_O) extract showed more intense peaks relating to HHDP-D-glucose (**1**) and to the two isomers of punicalagin (**5**, **6**).

### 3.3. ^1^H-NMR Analysis of P. granatum Juice and Extracts

**Figure 3:**
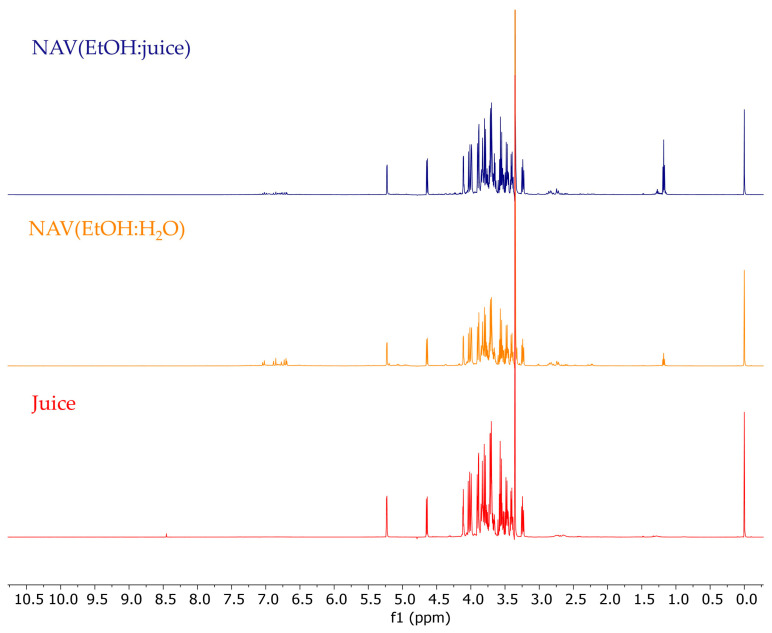
^1^H NMR spectra of *P. granatum* NAV(EtOH:juice) extract, NAV(EtOH:H_2_O) extract and juice.

To detect not only the specialised metabolites but also the primary metabolites present in juice, NAV(EtOH:H_2_O), and NAV(EtOH:juice) extracts, an approach based on ^1^H NMR analysis was carried out [39] (Figure 3). The non-destructive nature, simplicity of sample preparation, rapid analysis and avoidance of the need of chromatographic separations make ^1^H NMR a valuable technique in metabolite analysis [40].

**Figure 4:**
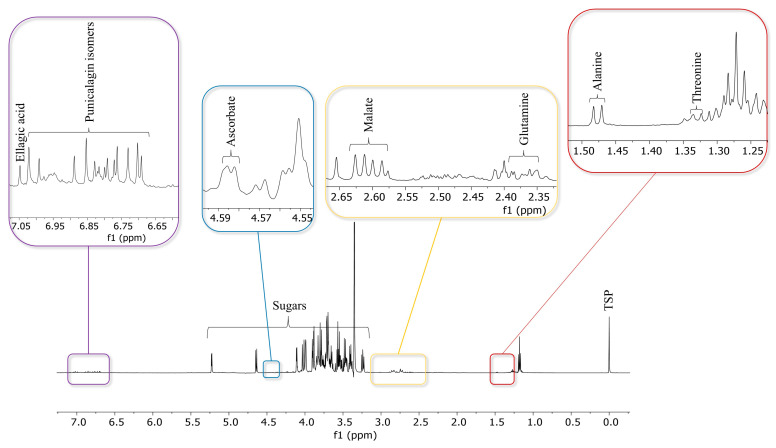
^1^H NMR spectrum of *P. granatum* NAV(EtOH:juice) extract.

This type of analysis is common in metabolomics and natural product studies, where the goal is to identify and quantify various compounds present in a complex mixture. The use of ^1^H NMR allows for a broad view of the sample composition. The identification of compounds was based on characteristic signals in the spectra. After an in-depth examination of ^1^H NMR spectra, the identified compounds, each with its own characteristic NMR signals, included amino acids, carbohydrates, ellagic acid, punicalagin isomers and ascorbate. A total of nine metabolites were identified by comparison with the literature data [41], the Chenomx and Human Metabolome Database, and based on standard two-dimensional NMR experiments (HSQC, HMBC and COSY) [20,42,43]. The identified metabolites are shown in Figure 4. The region from 3.00 to 5.00 ppm is typical of sugar proton (^1^H) signals. Signals corresponding to α and β glucose at 5.21 (d, *J =* 3.68 Hz) and 4.63 ppm (d, *J =* 7.62 Hz) and fructose at 4.09 ppm (d, *J =* 3.68 Hz) [44] were clearly evident.

The low-frequency region of the NMR spectrum provided valuable information about the presence of amino acids such as alanine at 1.46 ppm (d, *J =* 7.08 Hz), threonine at 1.31 ppm (d, *J =* 6.93 Hz) and glutamine (at 2.12 and 2.43 ppm, m) (Figure 4). Along with primary metabolites, the occurrence of specialised metabolites was revealed. Ellagic acid (**14**) (7.04 ppm, s) and punicalagin isomers (**5**, **6**) (eight singlets at 7.00, 6.97, 6.87, 6.83, 6.75, 6.74, 6.71, and 6.68 ppm) were identified in the aromatic region (Figure 4) [41].

The principal peak areas in the NMR spectrum are used as a measure of metabolite concentration. This implies that the intensity or area under specific peaks in the NMR spectrum corresponds to the amount of a particular metabolite. The quantification is performed based on a single internal standard, a substance of known concentration added to the sample to aid the quantification of other components. One of the common reference standards used in NMR spectroscopy for quantitative analysis is trimethylsilyl propionate (TSP). So, utilizing a known concentration of TSP as a reference, and employing the sophisticated targeted profiling technology provided by the Chenomx software (Chenomx NMR Suite version 10.0), the accurate deconvolution and quantification of identified metabolites was performed [45,46].

The metabolites average molar concentrations, expressed as mM, showed a high sugar content in pomegranate juice (glucose and fructose with a concentration of 16.994 and 20.477 mM, respectively), followed by NAV(EtOH:juice) extract (glucose and fructose with a concentration of 14.233 and 17.018 mM, respectively).

Regarding specialised metabolites, the NAV(EtOH:juice) extract appeared to be a good source of ellagic acid (0.129 mM).

Pomegranate juice is a highly recommended beverage with vitamin C, also known as ascorbic acid [2]. Vitamin C is required for the biosynthesis of collagen, L-carnitine, and certain hormones and neurotransmitters; it is also involved in protein metabolism and is an important physiological antioxidant [47,48]. In addition to its biosynthetic and antioxidant functions, vitamin C plays an essential role in immune function [49]. Insufficient vitamin C intake causes scurvy, which is characterised by fatigue or lassitude, widespread connective tissue weakness, and capillary fragility [47,48,49,50]. Moreover, due to its function as an antioxidant and its role in immune function, vitamin C prevents and/or treats numerous health conditions like cancer (including prevention and treatment), cardiovascular disease, age-related macular degeneration (AMD), cataracts and the common cold. The NAV(EtOH:juice) extract appeared enriched in vitamin C (0.309 mM), reported in Table 2 as ascorbate.

### 3.4. Total Phenolic, Tannin and Flavonoid Assays

The phenolic content evaluation was performed using the Folin–Ciocalteu method. The higher phenolic content in the NAV(EtOH:juice) extract (484.27 mg GAE/g) suggested that this combination of solvents (ethanol and juice) was more effective in extracting phenolic compounds compared to the NAV(EtOH:H_2_O) extract (114.10 mg GAE/g), which used ethanol and water.

A recent work reported the phenolic contents of ethanol extracts of pomegranate peels from eight different cultivars (ranging from 5766.44 to 10599.43 mg GAE/100 g) [29]. Moreover, the phenolic content reported for various extraction techniques from pomegranate peels resulted lower than that observed for the extract NAV (EtOH:juice) [51]. By comparing these data with our results, it is evident how the Naviglio extraction technique improved the total phenolic content and the choice of EtOH:juice as solvent positively affected the extraction efficiency. Pomegranate phenolics are useful as natural antioxidants for cosmeceutical applications for skin health, as evident in a recent in vitro study showing their protective effects against H_2_O_2_-induced oxidative stress and cytotoxicity in human keratinocyte HaCaT cells [52].

Also, the result of flavonoid content indicated a higher flavonoid content (432.33 mg rutin/g) in the extract NAV(EtOH:juice) than in the extract NAV(EtOH:H_2_O) (154.83 mg rutin/g). Regarding the estimation of tannin, in agreement with the results obtained from LC-MS and ^1^H-NMR analysis, the extract NAV(EtOH:H_2_O) showed the highest tannin (97.03 mg GAE/g) content (Table 3).

## 4. Conclusions

The growing consumption of pomegranate is motivated by the pleasant organoleptic properties and the beneficial constituents that make it a functional food. Besides this aspect, there is an increasing interest of consumers towards food supplements based on pomegranate which led to an increasing global pomegranate industry production. In recent years, new technologies and methods have been proposed for the development of green and environmentally friendly extraction. The literature data discussed before, including direct studies or trials that demonstrate these effects in vivo, enhance the credibility and applicability of the research. In this study, a new extraction procedure was developed to obtain a food supplement of *Punica granatum*, cultivar “Dente di Cavallo”, that was extracted, for the first time, through a non-conventional extraction technique, SLDE-Naviglio, using ethanol and pomegranate juice (70:30) as the extraction solvent. The NAV(EtOH:juice) extract showed an interesting chemical profile and a higher total phenolic content when compared with the juice and the peel extract obtained by SLDE-Naviglio using EtOH:H_2_O as the solvent. The enrichment of phenolics in the NAV(EtOH:juice) extract was evident in the LC-MS profiles of juice and extracts, revealing significant differences regarding the intensity of ellagic acid derivative and flavonoid peaks. Also NMR analysis which provided the quantification of some amino acids, carbohydrates, ellagic acid (**14**) and punicalagin isomers (**5**, **6**) showed higher amounts of these metabolites in NAV(EtOH:juice). It is noteworthy that the NMR analysis revealed the NAV(EtOH:juice) extract was enriched in vitamin C, which is essential in providing nutritional and therapeutic effects. This new extraction technique could be easily transferred to an industrial setting, just by modifying the drug:solvent ratio since the instrumentation of SLDE-Naviglio has different sizes. Laboratory models have an extraction chamber of 0.5-1-2 litres, while medium-sized models have an extraction chamber of 5-10-20-30-40 litres, and industrial-size models have an extraction chamber of 100-200-400 litres.

In conclusion, this work offers a new and feasible approach to enrich the juice of pomegranate by the use of pomegranate peels and to promote the Italian cultivar “Dente di Cavallo” as a source of food supplements.

## Figures and Tables

**Table 1:** Metabolites identified in *P. granatum* extracts by LC-ESI/QExactive/MS/MS analysis ^a^.

	R_t_	[M-H]^−^	[M-H]^2−^	Molecular Formula	Δppm	MS/MS	Name	NAV (EtOH:juice)	NAV (EtOH:H_2_O)	Juice
**1**	2.50	481.0625		C_20_H_18_O_14_	2.53	421.0427, 300.9991, 275.0199	HHDP-D-glucose	√	√	√
**2**	3.75	781.0542		C_34_H_22_O_22_	2.31	600.9899, 575.0115, 300.9988	Punicalin	√		√
**3**	5.09	649.0697		C_27_H_22_O_19_	2.48	498.5004, 300.9991	Lagerstannin C	√		√
**4**	7.44	305.0668		C_15_H_14_O_7_	3.09	287.0563, 219.0659, 179.0343, 165.0185, 137.0234, 125.0233	Epigallocatechin	√	√	
**5**	9.36	1083.0601	541.0262	C_48_H_28_O_30_	2.37	600.9901, 575.0101, 300.9991, 275.0199	Punicalagin isomer α	√	√	√
**6**	10.95	1083.0601	541.0262	C_48_H_28_O_30_	1.81	600.9901, 575.0101, 300.9991, 275.0199	Punicalagin isomer β	√	√	√
**7**	11.62	799.0646		C_34_H_24_O_23_	2.64	300.9991, 273.0043	Granatin A	√	√	√
**8**	12.99	785.0857		C_34_H_26_O_22_	3.17	300.9991, 275.0199, 249.0403	Pedunculagin II	√	√	√
**9**	13.02	633.0742		C_27_H_22_O_18_	3.03	463.0528, 419.0617, 300.9991, 275.0198	Galloyl-HHDP-hexoside	√	√	
**10**	13.54	463.0520		C_20_H_16_O_13_	3.83	300.9991, 275.0199, 249.0403	Ellagic acid glucoside	√	√	√
**11**	14.89	951.0748		C_41_H_28_O_27_	1.48	300.9991, 273.0043,	Granatin B	√	√	√
**12**	15.32	475.1824		C_21_H_32_O_12_	2.94	300.9984, 169.0133	Kanokoside A	√		√
**13**	15.98	433.0414		C_19_H_14_O_12_	2.83	300.9987	Ellagic acid pentose	√	√	
**14**	16.73	300.9991		C_14_H_6_O_8_	3.54	/	Ellagic acid	√	√	√
**15**	17.71	593.1523		C_27_H_30_O_15_	3.68	285.0404, 255.0298	Kaempferol 3-*O*-rutinoside	√		
**16**	18.55	447.0937		C_21_H_20_O_11_	3.36	301.0003, 284.0327, 255.0298	Astragalin	√		√
**17**	19.78	447.0939		C_21_H_20_O_11_	3.69	300.9984, 285.0405	Luteolin 4’-*O*-glucoside	√		√

^a^ The Rt and Δppm are referred to the profile of the butanol fraction of NAV(EtOH:juice) extract.

**Table 2:** Concentrations (expressed mM + SD) ^a^ of metabolites in *P. granatum* extracts and juice.

Metabolite	NAV (EtOH:juice)	NAV (EtOH:H_2_O)	Juice
Alanine	0.096 ± 0.004	0.046 ± 0.004	0.047 ± 0.001
Ascorbate	0.309 ± 0.047	0.233 ± 0.043	0.137 ± 0.009
Ellagic acid (**14**)	0.129 ± 0.007	0.066 ± 0.005	0.003 ± 0.001
Fructose	17.018 ± 0.482	10.677 ± 0.623	20.477 ± 0.506
Glucose	14.233 ± 0.637	11.435 ± 0.623	16.994 ± 0.414
Glutamine	0.128 ± 0.029	0.123 ± 0.024	0.070 ± 0.010
Malate	0.443 ± 0.050	0.266 ± 0.038	0.322 ± 0.019
Punicalagin isomers (**5**, **6**)	0.496 ± 0.036	1.133 ± 0.055	0.022 ± 0.004
Threonine	0.071 ± 0.013	0.028 ± 0.006	0.066 ± 0.006

^a^ SD: Standard deviation of three independent experiments.

**Table 3:** Total phenol, total tannin and total flavonoid content of *P. granatum* juice and extracts.

P. granatum	Total Phenol Content (mg GAE/g ± SD) ^a^	Total Flavonoid Content (mg rutin/g ± SD) ^a^	Total Tannin Content (mg GAE/g ± SD) ^a^
Juice	64.60 ± 2.52	268.17 ± 0.58	1.77 ± 1.02
NAV(EtOH:H_2_O)	114.10 ± 4.82	154.83 ± 0.25	97.03 ± 1.89
NAV(EtOH:juice)	484.27 ± 1.29	432.33 ± 1.59	71.47 ± 2.29

**^a^** SD: Standard deviation of three independent experiments.

## Data Availability

The original contributions presented in the study are included in the article and Supplementary Materials, further inquiries can be directed to the corresponding author.

## Supplementary Materials

The following supporting information can be downloaded at: https://www.mdpi.com/article/10.3390/foods13101429/s1, S1: ^1^H-NMR Analysis and Data Processing parameters; S2: Determination of Total Phenolic Content; S3: Determination of Total Flavonoid Content.

## References

[1] Yisimayili Z., Chao Z. (2022). A review on phytochemicals, metabolic profiles and pharmacokinetics studies of the different parts (juice, seeds, peel, flowers, leaves and bark) of pomegranate (*Punica granatum* L.). Food. Chem..

[2] Maphetu N., Unuofin J.O., Masuku N.P., Olisah C., Lebelo S.L. (2022). Medicinal uses, pharmacological activities, phytochemistry, and the molecular mechanisms of *Punica granatum* L. (pomegranate) plant extracts: A review. Biomed. Pharmacother..

[3] Borochov-Neori H., Judeinstein S., Tripler E., Harari M., Greenberg A., Shomer I., Holland D. (2009). Seasonal and cultivar variations in antioxidant and sensory quality of pomegranate (*Punica granatum* L.) fruit. J. Food Compos. Anal..

[4] Cruz-Valenzuela M.R., Ayala-Soto R.E., Ayala-Zavala J.F., Espinoza-Silva B.A., González-Aguilar G.A., Martín-Belloso O., Soliva-Fortuny R., Nazzaro F., Fratianni F., Tapia-Rodríguez M.R. (2022). Pomegranate (*Punica granatum* L.) Peel Extracts as Antimicrobial and Antioxidant Additives Used in Alfalfa Sprouts. Foods.

[5] Toledo-Merma P.R., Cornejo-Figueroa M.H., Crisosto-Fuster A.D.R., Strieder M.M., Chañi-Paucar L.O., Náthia-Neves G., Rodríguez-Papuico H., Rostagno M.A., Meireles M.A.A., Alcázar-Alay S.C. (2022). Phenolic Compounds Recovery from Pomegranate (*Punica granatum* L.) By-Products of Pressurized Liquid Extraction. Foods.

[6] Maghoumi M., Amodio M.L., Fatchurrahman D., Cisneros-Zevallos L., Colelli G. (2022). Pomegranate Husk Scald Browning during Storage: A Review on Factors Involved, Their Modes of Action, and Its Association to Postharvest Treatments. Foods.

[7] Rettig M.B., Heber D., An J., Seeram N.P., Rao J.Y., Liu H., Klatte T., Belldegrun A., Moro A., Henning S.M. (2008). Pomegranate extract inhibits androgen-independent prostate cancer growth through a nuclear factor-kappaB-dependent mechanism. Mol. Cancer Ther..

[8] An J., Guo Y., Wang T., Pantuck A.J., Rettig M.B. (2015). Pomegranate extract inhibits EMT in clear cell renal cell carcinoma in a NF-κB and JNK dependent manner. Asian J. Urol..

[9] Kim Y.E., Hwang C.J., Lee H.P., Kim C.S., Son D.J., Ham Y.W., Hellstrom M., Han S.B., Kim H.S., Park E.K. (2017). Inhibitory effect of punicalagin on lipopolysaccharide-induced neuroinflammation, oxidative stress and memory impairment via inhibition of nuclear factor-kappaB. Neuropharmacology.

[10] Li Z.P., Summanen P.H., Komoriya T., Henning S.M., Lee R.P., Carlson E., Heber D., Finegold S.M. (2015). Pomegranate ellagitannins stimulate growth of gut bacteria in vitro: Implications for prebiotic and metabolic effects. Anaerobe.

[11] Bellesia A., Verzelloni E., Tagliazucchi D. (2015). Pomegranate ellagitannins inhibit alpha-glucosidase activity in vitro and reduce starch digestibility under simulated gastro-intestinal conditions. Int. J. Food Sci. Nutr..

[12] Neyrinck A.M., Van Hée V.F., Bindels L.B., De Backer F., Cani P.D., Delzenne N.M. (2013). Polyphenol-rich extract of pomegranate peel alleviates tissue inflammation and hypercholesterolaemia in high-fat diet-induced obese mice: Potential implication of the gut microbiota. Br. J. Nutr..

[13] Rodriguez J., Gilson H., Jamart C., Naslain D., Pierre N., Deldicque L., Francaux M. (2015). Pomegranate and green tea extracts protect against ER stress induced by a high-fat diet in skeletal muscle of mice. Eur. J. Nutr..

[14] Middha S.K., Usha T., RaviKiran T. (2012). Influence of *Punica granatum* L. on region specific responses in rat brain during Alloxan-Induced diabetes. Asian Pac. J. Trop. Biomed..

[15] Murthy K.N.C., Jayaprakasha G.K., Singh R.P. (2002). Studies on Antioxidant Activity of Pomegranate (*Punica granatum*) Peel Extract Using in Vivo Models. J. Agric. Food Chem..

[16] Houston D.M.J., Bugert J., Denyer S.P., Heard C.M. (2017). Anti-inflammatory activity of *Punica granatum* L. (Pomegranate) rind extracts applied topically to ex vivo skin. Eur. J. Pharm. Biopharm..

[17] Hartman R.E., Shah A., Fagan A.M., Schwetye K.E., Parsadanian M., Schulman R.N., Finn M.B., Holtzman D.M. (2006). Pomegranate juice decreases amyloid load and improves behavior in a mouse model of Alzheimer’s disease. Neurobiol. Dis..

[18] Ropacki S.A., Patel S.M., Hartman R.E. (2013). Pomegranate Supplementation Protects against Memory Dysfunction after Heart Surgery: A Pilot Study. Evid.-Based Complement.Alternat. Med..

[19] Gadkari P., Daharwal S.J. (2022). Quantification of Punicalagin in Pomegranate Peels from High-performance Thin-layer Chromatography. Biomed. Biotechnol. Res. J..

[20] Abdul Hamid N.A., Mediani A., Maulidiani M., Abas F., Park Y.S., Leontowicz H., Leontowicz M., Namiesnik J., Gorinstein S. (2017). Characterization of metabolites in different kiwifruit varieties by NMR and fluorescence spectroscopy. J. Pharm. Biomed. Anal..

[21] Marra F., Petrovicova B., Canino F., Maffia A., Mallamaci C., Muscolo A. (2022). Pomegranate Wastes Are Rich in Bioactive Compounds with Potential Benefit on Human Health. Molecules.

[22] Arlotta C., Toscano V., Genovese C., Calderaro P., Puglia G.D., Raccuia S.A. (2022). Nutraceutical Content and Genetic Diversity Share a Common Pattern in New Pomegranate Genotypes. Molecules.

[23] Tarantino A., Difonzo G., Disciglio G., Frabboni L., Paradiso V.M., Gambacorta G., Caponio F. (2022). Fresh pomegranate juices from cultivars and local ecotypes grown in southeastern Italy: Comparison of physicochemical properties, antioxidant activity and bioactive compounds. J. Sci. Food Agric..

[24] Di Sotto A., Locatelli M., Macone A., Toniolo C., Cesa S., Carradori S., Eufemi M., Mazzanti G., Di Giacomo S. (2019). Hypoglycemic, Antiglycation, and Cytoprotective Properties of a Phenol-Rich Extract From Waste Peel of var. Dente di Cavallo DC2. Molecules.

[25] Deborde C., Moing A., Roch L., Jacob D., Rolin D., Giraudeau P. (2017). Plant metabolism as studied by NMR spectroscopy. Prog. Nucl. Magn. Reson. Spectrosc..

[26] Masullo M., Cerulli A., Mari A., de Souza Santos C.C., Pizza C., Piacente S. (2017). LC-MS profiling highlights hazelnut (Nocciola di Giffoni PGI) shells as a byproduct rich in antioxidant phenolics. Food. Res. Int..

[27] Cerulli A., Masullo M., Piacente S. (2021). Metabolite Profiling of *Helichrysum italicum* Derived Food Supplements by H-1-NMR-Based Metabolomics. Molecules.

[28] Chemat F., Vian M.A., Cravotto G. (2012). Green extraction of natural products: Concept and principles. Int. J. Mol. Sci..

[29] Peršurić Ž., Saftić Martinović L., Malenica M., Gobin I., Pedisić S., Dragović-Uzelac V., Kraljević Pavelić S. (2020). Assessment of the Biological Activity and Phenolic Composition of Ethanol Extracts of Pomegranate (*Punica granatum* L.) Peels. Molecules.

[30] Cano-Lamadrid M., Martínez-Zamora L., Castillejo N., Artés-Hernández F. (2022). From Pomegranate Byproducts Waste to Worth: A Review of Extraction Techniques and Potential Applications for Their Revalorization. Foods.

[31] Naviglio D., Scarano P., Ciaravolo M., Gallo M. (2019). Rapid Solid-Liquid Dynamic Extraction (RSLDE): A Powerful and Greener Alternative to the Latest Solid-Liquid Extraction Techniques. Foods.

[32] Parisi V., Santoro V., Donadio G., Bellone M.L., Diretto G., Sandri C., Mensitieri F., De Tommasi N., Dal Piaz F., Braca A. (2022). Comparative Chemical Analysis of Eight *Punica granatum* L. Peel Cultivars and Their Antioxidant and Anti-Inflammatory Activities. Antioxidants.

[33] Cerulli A., Napolitano A., Masullo M., Hošek J., Pizza C., Piacente S. (2020). Chestnut shells (Italian cultivar “Marrone di Roccadaspide” PGI): Antioxidant activity and chemical investigation with in depth LC-HRMS/MSn rationalization of tannins. Food. Res. Int..

[34] Moilanen J., Sinkkonen J., Salminen J.-P. (2013). Characterization of bioactive plant ellagitannins by chromatographic, spectroscopic and mass spectrometric methods. Chemoecology.

[35] Maldini M., Montoro P., Hamed A.I., Mahalel U.A., Oleszek W., Stochmal A., Piacente S. (2011). Strong antioxidant phenolics from *Acacia nilotica*: Profiling by ESI-MS and qualitative-quantitative determination by LC-ESI-MS. J. Pharm. Biomed. Anal..

[36] Akter S., Hong H., Netzel M., Tinggi U., Fletcher M., Osborne S., Sultanbawa Y. (2021). Determination of Ellagic Acid, Punicalagin, and Castalagin from Terminalia ferdinandiana (Kakadu plum) by a Validated UHPLC-PDA-MS/MS Methodology. Food Anal. Methods.

[37] Liu Y., Kong K.W., Wu D.-T., Liu H.-Y., Li H.-B., Zhang J.-R., Gan R.-Y. (2022). Pomegranate peel-derived punicalagin: Ultrasonic-assisted extraction, purification, and its α-glucosidase inhibitory mechanism. Food Chem..

[38] Abdulla R., Mansur S., Lai H., Ubul A., Sun G., Huang G., Aisa H.A. (2017). Qualitative Analysis of Polyphenols in Macroporous Resin Pretreated Pomegranate Husk Extract by HPLC-QTOF-MS. Phytochem. Anal..

[39] Cerulli A., Masullo M., Montoro P., Hosek J., Pizza C., Piacente S. (2018). Metabolite profiling of “green” extracts of *Corylus avellana* leaves by H-1 NMR spectroscopy and multivariate statistical analysis. J. Pharm. Biomed. Anal..

[40] Cerulli A., Masullo M., Pizza C., Piacente S. (2022). Metabolite Profiling of “Green” Extracts of *Cynara cardunculus* subsp. scolymus, Cultivar “Carciofo di Paestum” PGI by 1H NMR and HRMS-Based Metabolomics. Molecules.

[41] Kraszni M., Marosi A., Larive C.K. (2013). NMR assignments and the acid-base characterization of the pomegranate ellagitannin punicalagin in the acidic pH-range. Anal. Bioanal. Chem..

[42] Cicero N., Corsaro C., Salvo A., Vasi S., Giofre S.V., Ferrantelli V., Di Stefano V., Mallamace D., Dugo G. (2015). The metabolic profile of lemon juice by proton HR-MAS NMR: The case of the PGI Interdonato Lemon of Messina. Nat. Prod. Res..

[43] Kirmizibekmez H., Ariburnu E., Masullo M., Festa M., Capasso A., Yesilada E., Piacente S. (2012). Iridoid, phenylethanoid and flavonoid glycosides from *Sideritis trojana*. Fitoterapia.

[44] Borim de Souza A.J., Ocampos F.M.M., Catoia Pulgrossi R., Dokkedal A.L., Colnago L.A., Cechin I., Saldanha L.L. (2023). NMR-Based Metabolomics Reveals Effects of Water Stress in the Primary and Specialized Metabolisms of *Bauhinia ungulata* L. (Fabaceae). Metabolites.

[45] Corol D.I., Harflett C., Beale M.H., Ward J.L. (2014). An Efficient High Throughput Metabotyping Platform for Screening of Biomass Willows. Metabolites.

[46] Kılınc H., D’Urso G., Paolillo A., Alankus O., Piacente S., Masullo M. (2023). LC-MS and NMR Based Plant Metabolomics: A Comprehensive Phytochemical Investigation of *Symphytum anatolicum*. Metabolites.

[47] Li Y., Schellhorn H.E. (2007). New developments and novel therapeutic perspectives for vitamin C. J. Nutr..

[48] Carr A.C., Frei B. (1999). Toward a new recommended dietary allowance for vitamin C based on antioxidant and health effects in humans. Am. J. Clin. Nutr..

[49] Jacob R.A., Sotoudeh G. (2002). Vitamin C function and status in chronic disease. Nutr. Clin. Care.

[50] Frei B., England L., Ames B.N. (1989). Ascorbate is an outstanding antioxidant in human blood plasma. Proc. Natl. Acad Sci. USA.

[51] Lampakis D., Skenderidis P., Leontopoulos S. (2021). Technologies and Extraction Methods of Polyphenolic Compounds Derived from Pomegranate (*Punica granatum*) Peels. A Mini Review. Processes.

[52] Liu C., Guo H., DaSilva N.A., Li D.L., Zhang K., Wan Y.S., Gao X.H., Chen H.D., Seeram N.P., Ma H. (2019). Pomegranate *(Punica granatum*) phenolics ameliorate hydrogen peroxide-induced oxidative stress and cytotoxicity in human keratinocytes. J. Funct. Foods.

