# Supplementary material for: Development of a New Extraction Method for Pomegranate and Metabolite Profiling by a LC-MS and 1H NMR Combined Approach
Source: Foods. 2024 May 7;13(10):1429. doi: 10.3390/foods13101429 (PMC11120047; doi:10.3390/foods13101429)
Supplement: Supplementary file 1 [file foods-13-01429-s001.zip › foods-2979042-supplementary.pdf]

Supplementary Materials

Development of a New Extraction Method for Pomegranate and Metabolite Profiling by a LC-MS and ^{1}H NMR Combined Approach

S1. ^{1}H-NMR Analysis and Data Processing parameters

Number of transients	Dummy Scans	Data Points	Relaxation Delay
80	4	64k	5s

The collected spectra were automatically Fourier transformed using an exponential window with a line broadening of 0.5 Hz. Phase and baseline correction were performed using Chenomx NMR Suite 9.0 (Chenomx Inc., AB, Canada).

S2. Determination of Total Phenolic Content

The total phenolic content of the juice and extracts was determined using the Folin-Ciocalteu assay. As a standard reference, gallic acid was used. For the calibration curve, 30, 40, 50, 100, 200, 400, 600 and 800 μg/mL solutions of gallic acid were prepared and submitted to the assay following the same procedure used for the extracts. For gallic acid, the calibration equation was $y = 0.002x + 0.0168$ ($R^2 = 0.995$). All the experiments were performed in triplicate, and results were expressed as mean of gallic acid equivalents for gram of extract (GAE mg/g).

S3. Determination of Total Flavonoid Content

The total flavonoid content was measured using the Allumine Chloride colorimetric assay using rutin as a standard. A known volume of extract (1 mg/mL) was placed in a 10 mL volumetric flask.

Distilled water (5 mL) and a solution of $NaNO_2$ (1:20) (0.3 mL) were added. A solution of $AlCl_3$ (1:10) (3 mL) was added 5 min later. After 6 min, NaOH (1 M) (2 mL) and distilled water up to 10 mL were added. The solutions were mixed well, and the absorbance was measured against the blank control at 510 nm on a UV-visible spectrophotometer. Rutin was used as the standard for a calibration curve ($y = 0.0004x + 0.0554$, $R^2 = 0.985$). The content of flavonoids in the various extracts was expressed in rutin equivalents (RE).
